# Impact of collaborative chronic care management with remote monitoring on blood glucose and blood pressure in federally qualified health center patients: A pilot study

**DOI:** 10.1002/hsr2.1853

**Published:** 2024-02-07

**Authors:** Vincent Ekenga, Nedra Copelin, Clover Moten, Wylea Gray‐Winfrey, Christine C. Ekenga

**Affiliations:** ^1^ Xavier University of Louisiana College of Pharmacy New Orleans Louisiana USA; ^2^ EXCELth Inc. New Orleans Louisiana USA; ^3^ Rollins School of Public Health Emory University Atlanta Georgia USA

**Keywords:** chronic care management, diabetes, hypertension, remote monitoring, team‐based care

## 1 INTRODUCTION

Hypertension and type 2 diabetes are common conditions that affect more than 45% and 13% of United States adults, respectively.^1^, ^2^ Hypertension and diabetes are major risk factors for microvascular and macrovascular complications, such as cardiovascular disease. Adequate blood pressure and blood glucose control significantly decrease the development complications such as neuropathy, nephropathy, stroke, and myocardial infarction. Adequate blood pressure control is designated as less than 130/80 mmHg per the American College of Cardiology and a goal performance measure of less than 140/90 mmHg, according to the National Committee for Quality Assurance.^3^, ^4^ In terms of blood glucose control, the American Diabetes Association (ADA) recommends a glycemic target, hemoglobin A1c (A1c), of less than 7%.^5^ Given the high prevalence of both hypertension and diabetes, the ADA and US Center for Disease Control and Prevention promote the incorporation of team‐based care management for the treatment of these conditions.^6^, ^7^

In the United States, Federally Qualified Health Centers (FQHC) employ multiple specialties of healthcare professionals who emphasize the provision of coordinated care management to medically underserved populations.^8^ These populations often have high rates of chronic conditions and thus, may warrant additional services to improve health. Chronic care management (CCM) is a service in which primary care providers and clinical staff engage patients with two or more chronic conditions for additional care services. Clinic teams engage patients for at least 20 min per month with the aim of more coordinated care and improved outcomes.^9^ These CCM services can include pharmacist care, which has been shown to improve blood pressure and blood glucose among primary care patients.^10^, ^11^, ^12^ Pharmacists play a crucial role in improving blood pressure and blood glucose management by leveraging their expertise in medication management and patient education. Through medication reviews and counseling, pharmacists can enhance medication adherence and address potential barriers to treatment. Additionally, they can provide lifestyle counseling, emphasizing the importance of diet and exercise in controlling blood pressure and blood glucose levels. Collaborating with other healthcare providers, pharmacists contribute to comprehensive care, regularly monitoring patients and adjusting medications when necessary.

Multiple diabetes and hypertension practice guidelines recognize the utility of monitoring technologies for diagnosis, education, and management^3^, ^13^ However, limited access to these technologies pose significant barriers for groups who could benefit from remote monitoring solutions, including medically underserved populations such as FQHC patients. The purpose of this pilot study was to explore the impact of a team‐based (pharmacist and primary care provider) CCM approach, while incorporating remote monitoring technology, on the blood pressure and blood glucose of FQHC patients.

## 2 METHODS

### 2.1 Study design and participants

This study was conducted in four FQHCs in southeastern Louisiana, USA. Eligibility criteria included age ≥18 years old with uncontrolled type 2 diabetes mellitus (defined as hemoglobin A1C > 7%) or uncontrolled hypertension (defined as >140/90 mmHg). Eligible patients agreed to (1) use a study provided remote monitoring device, (2) complete monthly encounters with a pharmacist (in addition to regularly scheduled PCP appointments), and (3) provide written consent for study participation. Pregnant patients, patients with Type 1 Diabetes Mellitus, and patients using a continuous glucose monitoring device were excluded. This study was approved by the Institutional Review Board of Xavier University of Louisiana. Informed consent was obtained from every patient before study enrollment. A total of 29 patients were enrolled in the study between October 2020 and November 2021.

### 2.2 CCM management

Our study's CCM approach mirrored the approach supported by Medicare, a federally funded US insurance program that primarily covers individuals aged 65 years and older. Patients were referred to a pharmacist by a primary care provider, and patients encountered the pharmacist at least once a month. Pharmacists educated patients on device utilization, analyzed health data, and provided counseling regarding their chronic condition and medication therapy. If health goals were not met, the pharmacist collaborated with primary care providers to modify the treatment care plan or independently modified therapy.

### 2.3 Remote monitoring

Remote monitoring study devices and supplies were provided to all patients without cost.

A cellular‐enabled glucometer device was used to monitor blood glucose, and a Bluetooth‐ or cellular‐enabled device was used to monitor blood pressure.

### 2.4 Study outcomes

The main study outcome of interest was remote monitoring device use (yes/no) during the 3‐month period after the initial pharmacist consultation and beyond the initial 3‐month period (yes/no). Additional outcomes included change in (1) hemoglobin A1c (A1C), (2) mean in‐clinic systolic blood pressure, and (3) mean in‐clinic diastolic blood pressure and the relation of device usage to these measures.

### 2.5 Statistical analysis

Descriptive statistics were used to evaluate select study outcomes. Paired *t*‐tests were used to compare mean number of device readings before and after the pharmacist follow‐up visit. All tests were two‐sided and a *p*‐value of <0.05 was considered significant. All data analyses were conducted using SAS 9.4 (SAS Institute).

## 3 RESULTS

### 3.1 Diabetes

A total of 23 diabetes patients enrolled in the study. The sample mean age was 52 years. Diabetes patients were 57% women, 65% Non‐Hispanic Black, 17% Hispanic, 30% non‐English speaking, 61% publicly insured, and 30% uninsured. Approximately 91% (21/23) used the glucometer during the initial 3‐month period, and only 47% (11/23) used the device after the initial 3‐month period (Table 1). Overall, there were nonsignificant decreases in A1C (*p* = 0.17). A1C levels decreased by 0.8% (*p* = 0.28) among the 21 patients that used the glucometer during the initial 3‐month period. A1C levels decreased by 1.6% (*p* = 0.11) among those patients with longer term use after the initial 3‐month period.

**Table 1:** Characteristics of diabetes patients by usage group (*n* = 23).

Characteristic	Total *n* = 23	Usage within first 3 months *n* = 21	Usage after first 3 months *n* = 11	*p*‐Value^a^
Total weeks of usage, mean (SD)^b^	10.8 (9.2)	10.8 (9.2)	15.7 (10.2)	0.17
First A1c, mean % (SD)	10.7 (2.1)	10.7 (2.2)	11.0 (2.5)	0.71
Final A1c, mean % (SD)	9.8 (2.3)	9.9 (2.4)	9.4 (2.1)	0.54
∆A1c, mean % (SD)	−0.9 (0.5)	−0.8 (0.05)	−1.6 (1.9)	0.44
*p*‐Value comparing first to final A1c	0.17	0.28	0.11	
Age, mean years (SD)	52.3 (11.2)	50.5 (9.3)	52.6 (9.0)	0.55
BMI, mean (SD)	32.1 (9.2)	32.9 (9.2)	33.5 (11.4)	0.87
Comorbidities, mean (SD)	3.2 (1.1)	2.0 (1.0)	2.3 (1.0)	0.54
Gender (*n* [%])				
Male	10 (43.5)	9 (42.9)	3 (27.3)	0.23
Female	13 (56.5)	12 (57.1)	8 (72.7)	
Race (*n* [%])				0.34
Non‐Hispanic Black	15 (65.2)	14 (66.7)	5 (45.5)	
Hispanic other	4 (17.4)	4 (19.0)	3 (27.3)	
Non‐Hispanic other	4 (17.4)	3 (14.2)	1 (9.1)	
Primary language (*n* [%])				0.44
English	16 (69.6)	13 (61.9)	6 (54.5)	
Spanish	5 (21.7)	5 (23.8)	4 (36.4)	
Arabic	1 (4.3)	2 (9.5)	0 (0.0)	
Creole	1 (4.3)	1 (4.8)	1 (9.1)	
Insurance status (*n* [%])				0.44
Medicaid	12 (52.2)	12 (57.1)	6 (54.5)	
Dual eligible	2 (8.7)	0 (0.0)	0 (0.0)	
Self‐pay/uninsured	7 (30.4)	7 (33.3)	4 (36.4)	
Private insurance	2 (8.7)	2 (9.5)	1 (9.1)	
BMI (*n* [%])				0.09
30+	12 (52.2)	12 (57.1)	6 (54.5)	
<30	11 (47.8)	9 (42.9)	5 (36.4)	
Comorbidities (*n* [%])				0.48
0	1 (21.4)	1 (4.8)	0 (0.0)	
1	3 (21.4)	3 (14.3)	1 (9.1)	
2	14 (35.7)	13 (61.9)	8 (72.7)	
3	3 (21.4)	3 (14.3)	1 (9.1)	
5	2 (21.4)	1 (4.8)	1 (9.1)	
Insulin therapy (*n* [%])				0.23
Yes	15 (65.2)	8 (38.1)	7 (63.6)	
No	8 (34.8)	13 (61.9)	4 (36.4)	

^a^ *p*‐Value for *t*‐test comparing the two usage groups.

^b^ Excluded 2 who never used device.

### 3.2 Hypertension

A total of six hypertension patients enrolled in the study. The sample mean age was 63 years. Hypertension patients were 50% women, 100% Non‐Hispanic Black, 20% non‐English speaking, and 100% publicly insured. The mean BMI was 31.9 (SD = 10.8) kg/m^2^. Half (3/6) used the blood pressure monitor during the initial 3‐month period, and none of the patients used the device after. Among those that used the monitor during the initial 3 months, the average systolic change was a nonsignificant 4.9 mmHg decrease (*p* = 0.47) and the average diastolic change was 1.06 mmHg (*p* = 0.80) (Table 2). Among the participants that did not use the blood pressure monitor, there was an average systolic increase of 0.33 mmHg (*p* = 0.97) and the average diastolic decrease was 0.33 mmHg (*p* = 0.96).

**Table 2:** Hypertension patient characteristics, device usage, and mean blood pressure at baseline and end of follow‐up (*n* = 6).

Characteristic	Total *n* = 6	Device usage YES *n* = 3	Device usage NO *n* = 3	*p*‐Value^a^
Age, mean years (SD)	63.3 (19.3)	53.3 (23.0)	73.3 (10.1)	0.24
Comorbidities, mean (SD)	3.3 (1.0)	3.3 (0.6)	3.3 (1.5)	0.99
Systolic blood pressure				
Baseline mean (SD), mm Hg	151.8 (17.7)	143.7 (15.8)	154.5 (20.1)	
Follow‐Up mean (SD), mm Hg	146.8 (15.0)	136.2 (7.5)	154.8 (14.0)	
∆Systolic blood pressure (SD), mm Hg	−4.9 (6.7)	−7.5 (7.1)	0.33 (10.0)	
*p*‐Value comparing baseline to follow‐up	0.47	0.32	0.97	
Diastolic blood pressure				
Baseline mean (SD), mm Hg	79.1 (11.4)	82.5 (6.9)	81.2 (14.7)	
Follow‐Up mean (SD), mm Hg	78.1 (12.9)	80.2 (16.2)	80.8 (7.8)	
∆Diastolic blood pressure (SD), mm Hg	−1.06 (4.1)	−2.3 (7.2)	−0.33 (6.8)	
*p*‐Value comparing baseline to follow‐up	0.80	0.75	0.96	

^a^ *p*‐value for *t*‐test comparing the two usage groups.

## 4 DISCUSSION

This study examined the impact of team‐based CCM, while utilizing remote monitoring, on hypertension and type 2 diabetes among FQHC patients. Study findings indicate that extended care management with a pharmacist and primary care provider team may have a positive impact on blood pressure and blood glucose. This positive impact was further realized when patients engaged with remote monitoring technology, as increased length of engagement with technology was associated with greater reductions (−1.6% blood glucose and 7.5/2.3 mmHg blood pressure). The results obtained were not found to be statistically significant, but may be of clinical significance.

CCM's positive effect on A1C reduction and blood pressure is consistent with previous studies. A meta‐analysis by Elissen and associates that examined the effectiveness of CCM found a statistically significant change in A1C (–0.5%) and a significant overall reduction in systolic blood pressure of 2.8 mmHg.^14^ Elissen et al. also found that the most promising results were found in studies with a shorter follow‐up period (1 year or less),^14^ similar to our study's follow‐up period of less than 1 year. The involvement of pharmacists in CCM may also positively influence blood pressure and blood glucose as study pharmacists were authorized to modify pharmacotherapy treatment regimens and remained in constant communication with primary care providers. To maximize continuity of care, pharmacists made therapeutic interventions after collaborating with primary care providers or independently as deemed necessary. Pharmacists also addressed other patient concerns including refill authorizations, prior authorizations, referrals to satisfy care gaps, reviewed care transitions, and analyzed all medication regimens for appropriateness, efficacy, safety, and adherence. Our results support prior findings that authorizing nonphysician providers to initiate and intensify therapy may decrease clinical inertia and improve A1C.^15^

Remote monitoring, a method utilizing technological devices to transmit patient vitals to health care providers from a distance, is often combined with telehealth services such as patient care and education.^16^ Like CCM, remote monitoring can improve blood glucose and blood pressure. A meta‐analysis by Zhu and colleagues analyzed 20 trials and found that remote monitoring significantly reduced A1C by 0.42%. They also found nonclinically significant reductions in systolic and diastolic blood pressure, 0.10 and 0.07 mmHg respectively.^17^ Similar to previous studies, we observed better outcomes among patients who engaged with remote monitoring technologies. However, our results were limited by sample size, and larger studies will be required to further assess the impact of combining CCM with remote monitoring technologies. Given recent advances in artificial intelligence (AI),^18^, ^19^ and AI's potential to analyze vast amounts of patient data collected through remote monitoring, further large‐scale studies on the impacts of remote monitoring on patient outcomes are warranted.

In the United States, CCM is a covered by Medicare, but is inconsistently provided among private payers or Medicaid, a government funded insurance program that primarily covers low‐income individuals. Given the demographics of our study population, our results indicate that there is possible benefit of CCM to multiple populations other than the traditionally eligible (age 65+ years) Medicare patients. CCM with remote monitoring may also beneficial to a broad spectrum of patients, emphasizing a rationale for service expansion in primary care and across insurers.

## 5 STRENGTHS AND LIMITATIONS

Strengths of the study include the recruitment of underserved patients from FQHCs. However, we acknowledge that sample size is a limitation to the interpretation of study, particularly for interpreting blood pressure outcomes. Our sample size was the result of a limited number of primary care provider referrals to pharmacists during the study period, further highlighting the need to create more team‐based care opportunities. Another limitation was the use of in‐clinic A1c and blood pressure values as endpoints, instead of remote monitored values as endpoints. In the United States, in‐clinic values are submitted by organizations to accrediting bodies for performance evaluations. Therefore, to parallel those reporting practices, our study focused on in‐clinic endpoints. Finally, our study recruited patients from FQHCs, and it is important to note the challenges associated with remote monitoring of lower‐income patients. We encountered potential study participants with incompatible or older phones, slow or limited cellular service, and limited technology literacy. These challenges prompted our study to transition to fully automated and cellular‐enabled monitoring devices. However, we believe this adjustment was a strength of our study as it allowed us to enroll patients with more diverse experiences, including low‐income patients and patients with low‐tech literacy.

## 6 CONCLUSIONS

Achieving adequate blood pressure and blood glucose control is instrumental in maintaining overall health and preventing complications for those with hypertension and diabetes. Team‐based (pharmacist and primary care provider) CCM with remote monitoring may positively impact blood pressure and blood glucose among FQHC patients. Engaging patients with remote technology for extended periods may contribute to better health outcomes. Further study with longer follow‐up is necessary to confirm the consistency of impact and inform ideal technical specifications for underserved patient populations.

## AUTHOR CONTRIBUTIONS

**Vincent Ekenga**: Conceptualization; funding acquisition; investigation; methodology; project administration; supervision; writing—original draft; writing—review and editing. **Nedra Copelin**: Data curation; investigation; writing—review and editing. **Clover Moten**: Data curation; investigation; writing—review and editing. **Wylea Gray‐Winfrey**: Investigation; supervision; writing—review and editing. **Christine C. Ekenga**: Data curation; investigation; methodology; visualization; writing—original draft; writing—review and editing. All authors have read and approved the final version of the manuscript.

## CONFLICT OF INTEREST STATEMENT

The authors declare no conflict of interest.

## ETHICS STATEMENT

All patients in this manuscript have given written informed consent for participation in the study and the use of their deidentified, anonymized, aggregated data and their case details (including photographs) for publication. This study was approved by the Institutional Review Board of Xavier University of Louisiana (certificate #806).

## TRANSPARENCY STATEMENT

The corresponding author Christine C. Ekenga affirms that this manuscript is an honest, accurate, and transparent account of the study being reported; that no important aspects of the study have been omitted; and that any discrepancies from the study as planned (and, if relevant, registered) have been explained.

## Data Availability

The data that support the findings of this study are available from the corresponding author upon reasonable request. Christine Ekenga had full access to all of the data in this study and takes complete responsibility for the integrity of the data and the accuracy of the data analysis.
